# Correction: Bustamante et al. Impact of Potassium Pre-Harvest Applications on Fruit Quality and Condition of Sweet Cherry (*Prunus avium* L.) Cultivated under Plastic Covers in Southern Chile Orchards. *Plants* 2021, *10*, 2778

**DOI:** 10.3390/plants12112108

**Published:** 2023-05-26

**Authors:** Marco Bustamante, Ariel Muñoz, Iverly Romero, Pamela Osorio, Sergio Mánquez, Rocío Arriola, Marjorie Reyes-Díaz, Alejandra Ribera-Fonseca

**Affiliations:** 1 Centro de Fruticultura, Facultad de Ciencias Agropecuarias y Forestales, Campus Andrés Bello, Universidad de La Frontera, Avenida Francisco Salazar 01145, Temuco P.O. Box 24-D, Chile; marco.bustamante@ufrontera.cl (M.B.); ariel.munoz@ufrontera.cl (A.M.); 2 Instituto de Investigaciones Agropecuarias (INIA), Carillanca Station, km 10 Camino Cajón-Vilcún, Temuco P.O. Box 929, Chile; iverlyromero@gmail.com; 3 Research, Development and Innovation Department, Exportadora Rancagua S.A.—Ranco Cherries, Route 5 South, 04000, km 80, Rancagua P.O. Box 576, Chile; posorio@ranco.cl (P.O.); smanquez@ranco.cl (S.M.); rarriola@ranco.cl (R.A.); 4 Departamento de Ciencias Químicas y Recursos Naturales, Facultad de Ingeniería y Ciencias, Campus Andrés Bello, Universidad de La Frontera, Avenida Francisco Salazar 01145, Temuco P.O. Box 24-D, Chile; marjorie.reyes@ufrontera.cl; 5 Center of Plant-Soil Interaction and Natural Resources Biotechnology, Scientific and Technological Bioresource Nucleus (BIOREN), Campus Andrés Bello, Universidad de La Frontera, Avenida Francisco Salazar 01145, Temuco P.O. Box 24-D, Chile

In the original publication [1], there was a mistake in Table 3 as published. The names of five fruit parameters included in the table (weight, caliber, TSS, TA, and maturity index) were not correct. Furthermore, the data did not correspond to the respective fruit parameters, except for ‘firmness’ (the first parameter). The corrected Table 3 appears below.

The authors apologize for any inconvenience caused and state that the scientific conclusions are unaffected. This correction was approved by the Academic Editor. The original publication has also been updated.

## Figures and Tables

**Table 3:** Quality and condition parameters at post-harvest in fruits of sweet cherry (cultivar Regina) cultivated at two commercial orchards of southern Chile (Perquenco and Puerto Octay). In each orchard, trees were subjected to two foliar potassium (K) treatments: conventional K regime (four sprays during the season; K− treatment) or intensive K regime (seven sprays during the season; K+ treatment). In each location, the assays were conducted in two consecutive seasons. In Perquenco orchard, covered and un-covered trees were compared.

Orchard	Season	Fruit Parameter	Treatments			
			Covered		Un-Covered	
			K−	K+	K−	K+
Perquenco	2019	Firmness (g/mm)	327 ± 18 Db	369 ± 18 Cb	418 ± 22 Bb	471 ± 18 Aa
	2020		468 ± 14 Ba	458 ± 34 Ba	493 ± 42 Aa	479 ± 20 Aa
Puerto Octay	2020		476 ± 49 Aa	441 ± 24 Aa		
	2021		281 ± 20 Bc	325 ± 23 Ac		
Perquenco	2019	TSS (Brix)	16.3 ± 0.9 Bb	18.4 ± 1.2 Aab	16.6 ± 0.7 Bb	18.1 ± 0.5 Ab
	2020		19.5 ± 0.9 Ba	19.6 ± 1.2 ABa	20.2 ± 2.0 ABa	21.1 ± 1.6 Aa
Puerto Octay	2020		19.9 ± 1.4 Aa	19.1 ± 1.3 Aa		
	2021		15.8 ± 1.1 Bb	17.3 ± 1.4 Ab		
Perquenco	2019	TA (% malic acid)	0.31 ± 0.02 Bc	0.36 ± 0.04 ABb	0.33 ± 0.02 Bb	0.41 ± 0.03 Ab
	2020		0.58 ± 0.09 Ba	0.53 ± 0.05 Ba	0.61 ± 0.06 Aa	0.59 ± 0.02 Aa
Puerto Octay	2020		0.37 ± 0.01 Ab	0.38 ± 0.04 Ab		
	2021		0.39 ± 0.17 Aab	0.37 ± 0.01 Ab		
Perquenco	2019	Cracking (%)	0.4 ± 0.2 Bb	0.8 ± 0.2 Bc	10.7 ± 2.6 Aa	9.2 ± 3.3 Aa
	2020		1.2 ± 0.6 Bb	1.9 ± 1.0 Bab	14.7 ± 5.2 Aa	12.4 ± 3.7 Aa
Puerto Octay	2020		2.9 ± 0.7 Aa	2.8 ± 1.3 Aa		
	2021		2.1 ± 1.2 Aab	1.2 ± 0.2 Ab		
Perquenco	2019	Pedicel Browning (%)	5.0 ± 1.0 Cb	5.7 ± 1.5 Cc	11.7 ± 1.5 Ba	17.3 ± 3.8 Aa
	2020		4.7 ± 1.2 Bb	5.3 ± 2.4 Bc	8.1 ± 1.9 Ab	9.5 ± 3.5 Ab
Puerto Octay	2020		9.3 ± 3.2 Ba	14.4 ± 4.9 Ab		
	2021		14.5 ± 3.8 Ba	24.0 ± 5.9 Aa		
Perquenco	2019	Pitting (%)	16.3 ± 1.0 Aa	4.9 ± 2.5 Bb	7.4 ± 2.7 Aba	1.6 ± 0.2 Bb
	2020		14.1 ± 5.2 Aab	7.3 ± 3.8 Bab	5.4 ± 2.8 Bca	3.1 ± 1.1 Ca
Puerto Octay	2020		12.3 ± 5.4 Ab	8.7 ± 3.6 Ba		
	2021		16.3 ± 1.0 Aa	5.0 ± 2.5 Bb		

For each parameter, average values ± standard deviation are presented. Statistically significant differences between treatments, for each season and orchard, are represented by different uppercase letters (horizontally). Differences between season and orchard, for each K treatment, are represented by vertical lowercase letters based on the LSD Fisher multiple range test (*p* ≤ 0.05).
